# Generation of functionally distinct isoforms of PTBP3 by alternative splicing and translation initiation

**DOI:** 10.1093/nar/gkv429

**Published:** 2015-05-04

**Authors:** Lit-Yeen Tan, Peter Whitfield, Miriam Llorian, Elisa Monzon-Casanova, Manuel D. Diaz-Munoz, Martin Turner, Christopher W.J. Smith

**Affiliations:** 1 Department of Biochemistry, University of Cambridge, Tennis Court Road, Cambridge CB2 1QW, UK; 2 Laboratory of Lymphocyte Signalling and Development, The Babraham Institute, Babraham Research Campus, CB22 3AT, UK

## Abstract

Polypyrimidine tract binding protein (PTBP1) is a widely expressed RNA binding protein that acts as a regulator of alternative splicing and of cytoplasmic mRNA functions. Vertebrates contain two closely-related paralogs with >75% amino acid sequence identity. Early replacement of PTBP1 by PTBP2 during neuronal differentiation causes a concerted set of splicing changes. By comparison, very little is known about the molecular functions or physiological roles of PTBP3, although its expression and conservation throughout the vertebrates suggest a role in haematopoietic cells. To begin to understand its functions we have characterized the mRNA and protein isoform repertoire of PTBP3. Combinatorial alternative splicing events at the 5′ end of the gene allow for the generation of eight mRNA and three major protein isoforms. Individual mRNAs generate up to three protein isoforms via alternative translation initiation by re-initiation and leaky scanning using downstream AUG codons. The N-terminally truncated PTBP3 isoforms lack nuclear localization signals and/or most of the RRM1 domain and vary in their RNA binding properties and nuclear/cytoplasmic distribution, suggesting that PTBP3 may have major post-transcriptional cytoplasmic roles. Our findings set the stage for understanding the non-redundant physiological roles of PTBP3.

## INTRODUCTION

RNA binding proteins are the key players that orchestrate regulation of gene expression at post-transcriptional levels, including nuclear pre-mRNA processing, mRNA transport to the cytoplasm, localization, translation and turnover. RNA binding proteins are often not dedicated to a single molecular process but can regulate many of the steps in the mRNA life-cycle. Consequently, a characteristic feature of many of these multi-tasking proteins is the ability to shuttle between the nucleus and the cytoplasm. Some of these regulatory proteins are members of close families of paralogs, the members of which show a high degree of redundancy in function but, crucially, which also have differential activity on subsets of transcripts. Cell-type specific switching between expression of different paralogs can be used to regulate programs of post-transcriptional gene expression (reviewed in (1)). A good example of a family of nuclear and cytoplasmic multi-tasking proteins is provided by mammalian Polypyrimidine Tract Binding protein (PTBP1 also known as PTB) and its paralogs PTBP2 (also known as neuronal or brain PTB: nPTB/brPTB) and PTBP3 (also known as Regulator of Differentiation 1: ROD1).

PTBP1 was originally identified as a protein that could bind to the polypyrimidine tract at the 3′ splice site of introns (2,3). It was subsequently shown to be a repressive regulator of splicing (4,5), although more recent global analyses show that it can both repress or activate splicing dependent on its location of binding (6–9). PTBP1 also regulates pre-mRNA 3′ end processing and various cytoplasmic functions, including mRNA localization, stability and translation (10,11). Although the steady-state distribution of PTBP1 in many cells is predominantly nuclear, protein kinase A phosphorylation of Ser-16, which is embedded within a bipartite nuclear localization signal (NLS) leads to a redistribution towards the cytoplasm allowing PTBP1 to regulate cytoplasmic functions (12,13). Much is known about the role of switching between PTBP1 and PTBP2 expression during neuronal differentiation and maturation (reviewed in (14)). In non-neuronal cells, PTBP2 expression is switched off, even if the gene is transcribed, by PTBP1 induced skipping of PTBP2 exon 10, which leads to Nonsense Mediated Decay (15–17). During neuronal differentiation, miR124 downregulates PTBP1 expression, which in turn leads to upregulation of PTBP2 (16). Although the PTBP2 has similar activity to PTBP1 for many events (17), a coordinated set of splicing events are sensitive to the switch from PTBP1 to PTBP2 (15). Later in development, PTBP2 expression also decreases and this in turn leads to a second wave of alternative splicing changes characteristic of adult brain and essential for brain development (8,18).

While the functions and developmental roles, particularly in neurons, of PTBP1 and PTBP2 have been well studied the third mammalian paralog, PTBP3 has been relatively neglected. Originally identified as Regulator of Differentiation 1 (ROD1 (19)) it has been identified primarily in haematopoietic cells. By analogy with the key role of PTBP2 in neuronal differentiation, we expect that PTBP3 will have an important non-redundant role in the hematopoietic system. Given the high degree of amino acid identity of PTBP3 with PTBP1 and 2, it is expected to be involved in similar molecular functions. Splicing repressor activity of PTBP3 has been demonstrated upon a *FAS* exon 6 splicing reporter (20) while analysis of interacting proteins indicated an unexpected involvement with Nonsense Mediated Decay (21). PTBP1 and PTBP2 also cause skipping of exon 2 of PTBP3, but the functional consequences of this event have been unclear (17). When exon 2 is included, AUG1 (i.e. the first AUG triplet from the 5′ end of the transcript; see Figure 2 legend for explanation of AUG numbering) in exon 1 opens a reading frame encoding a 555-amino acid protein with 76% identity to PTBP1 (Supplementary Figure S1). Skipping of the 34 nt exon 2 causes a frameshift and termination of the AUG1 reading frame after only 20 codons. Despite the fact that exon 2 skipped mRNAs have the upstream ORF (uORF) initiating at AUG1, they are not substrates for Nonsense Mediated Decay (NMD) (17). The annotated start codon for transcripts lacking exon 2 (e.g. ENST00000374257) is AUG4 (19). Indeed, western blot detection of PTBP3 showed proteins of ∼57 and 50 kDa, the larger of which could correspond to protein initiating at AUG4 (19). However, the identity of the 50 kDa band is unclear and no protein of ∼60 kDa, corresponding to initiation at AUG1, was detected.

**Figure 1.**
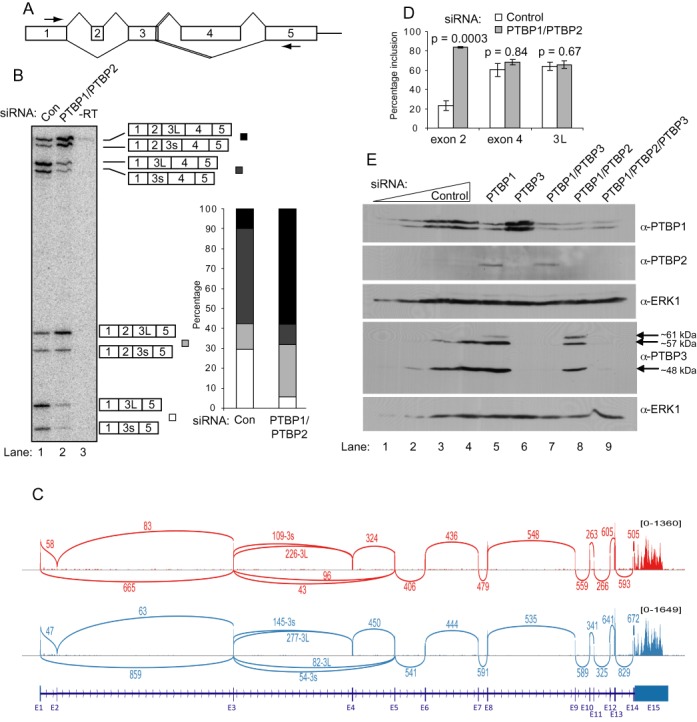
Combinatorial alternative splicing of PTBP3. (**A**) Schematic of exon structure and alternative splicing events at 5′ end of PTBP3. Arrows indicate location of PCR primers used in (**B**). (B) RT-PCR of PTBP3 mRNA in K562 cells using PCR primers in exons 1 and 5 after treatment with control siRNA (lane 1) or PTBP1/PTBP2 siRNA (lane 2). Cartoon schematics to the right indicate the identity of the 8 bands. 3s and 3L denote the use of the upstream and downstream alternative 5′ ss of exon 3. The histogram to the right indicates the proportions of mRNA isoforms with exon 2 and/or exon 4 skipped. (**C**) Sashimi plots showing RNAseq data of K562 cells. Data from two RNAseq libraries are shown. Numbers above arcs indicate the number of reads found that map to a particular exon–exon junction. (**D**) Quantitation of data from (B) to indicate the percentage inclusion of exon 2, exon 4 and the 3L 5′ ss. Only exon 2 inclusion is sensitive to PTBP1/PTBP2 knockdown. (**E**) Effects of siRNAs targeting PTBP1, 2 and 3, labelled here above lanes as P1, P2 and P3 respectively. Western blots used the antibodies indicated to the right; α-PTBP1 (BB7) and α-PTBP3-FL. Lanes, 3, 2, 1: two-fold dilutions of the control siRNA treated sample in lane 4.

**Figure 2.**
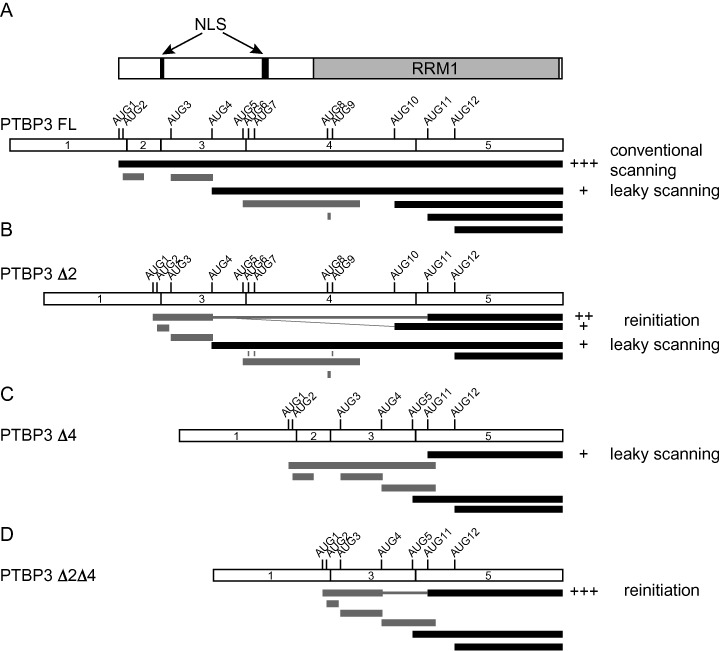
Summary of translation initiation from PTBP3 isoforms. Each panel schematically shows one of the four main PTBP3 mRNA isoforms (all with 3L) above, with potential open reading frames (ORFs) below. (**A**) PTBP3 FL mRNA, with the schematic domain organization of the encoded protein above (**B**) PTBP3 Δ2 with exon 2 skipped, (**C**) PTBP3 Δ4 with exon 4 skipped, (**D**) PTBP3 Δ2Δ4 with exons 2 and 4 skipped. Black bars represent ORFs that encode full length PTBP3 isoforms. Grey bars represent uORFs. AUG codons are numbered and indicated according to their relative location from the 5′ end of the full length PTBP3 isoform. Note that the numbering of AUGs refers to the full length isoform, even though AUGs 6–10 lie within exon 4 and are missing from the exon 4 skipped isoforms. The annotations to the right and scores (+, ++ and +++) indicate the relative levels of translation from each AUG and inferred mechanism from data in Figure 3. Connectors between uORFs and main ORFs imply translational reinitiation.

Given its expected importance in haematopoietic cells, we set out to characterize the mRNA and protein isoform diversity of PTBP3. We show that complex AS at the 5′ end of *PTBP3* results in the generation of 8 distinct mRNA isoforms, none of which is NMD sensitive. However, the generation of uORFs results in individual mRNA isoforms that generate more than one protein isoform via translation initiation at downstream AUGs by mechanisms of re-initiation and leaky scanning. The resultant PTBP3 protein isoforms are truncated at the N-terminus, differ in the presence of the bipartite NLS and number of intact RNA Recognition Motif (RRM) domains, and in their subcellular localization and RNA binding. Strikingly, a major protein isoform initiates at AUG11, truncating RRM1; this major initiation codon has not been correctly identified in the annotated Refseq, Ensembl, or Gencode isoforms.

## MATERIALS AND METHODS

### DNA constructs

All PTBP3 expression constructs used the pEGFP-N1 vector. To study the expression of PTBP3 mRNAs, two I.M.A.G.E. clones were used for full length PTBP3 (CR749471) and PTBP3Δ2 (BC039896). The PTBP3Δ4 and PTBP3Δ2Δ4 ORFs were cloned from cDNAs extracted from K562 cells. Mutations to study translation initiation used AUG to UUG mutations to disrupt recognition of initiator codons (UUG), tgggccAUGga to gccaccAUGga mutation to put AUG1 in a perfect Kozak context (cAUG) and UGA to UGC mutation of the stop codon in the AUG1 uORF to allow expression from AUG1 (fAUG). The FAS exon 6 splicing reporter containing the human genomic sequence from exons 5 to 7, was kindly provided by K. M. Izquierdo and J. Valcarcel. A codon optimized AUG1 PTBP3 ORF sequence for transient expression in mammalian cells was obtained from GeneArt and was cloned into HindIII/BamHI sites of the pEGFP-N1 vector (Clontech). We cloned sequences starting from AUG4 and AUG11 to force expression from these start codons. To direct nuclear expression of PTBP3 isoforms, the SV40 NLS PKKKRKV was cloned into the vectors into BamHI/AgeI sites. PTB1-GFP and PTB4-GFP expression vectors were generated in pEGFP-N1. For expression of C-terminally His-tagged recombinant PTBP3 proteins, the PTBP3 ORF sequences were cloned into the pET-21d vector using EcoRI/HindIII sites.

### Cell culture and transfections

K562 cells were maintained in 10 cm plates in RPMI medium with glutamax (Life Technologies) supplemented with 10% (v/v) Fetal Calf Serum, Biosera (FCS). RNAi transfections in K562 cells were performed as follows. On day 1, K562 cells were seeded in spinner flasks at 2 × 10^5^ cells/ml. On day 2, 2 × 10^5^ cells were seeded in 24-well plates in 100 μl medium. A total of 20 pmol siRNA (5 pmol P1, 10 pmol N1, 5 pmol Rd9 made up with C2) was diluted in 100 μl OptiMEM-1 (Gibco, Life technologies), then 6 μl HiPerfect (QIAGEN) was added and the mixture incubated for 10 min. This transfection mixture was added to each well, then after 6 h, 400 μl RPMI was added to each well. On day 3, cells were transferred to a 6-well plate and the siRNA transfection was repeated. After 6 h, 1.5 ml RPMI was added to each well. Cells were harvested after 72 h. mRNA targets for gene specific knockdown were as follows: human PTBP1 P1 (22); human PTBP2 N1 (17), human PTBP3 Rd9, 5′-UGCCGUUACUAUGGUGAAUUA-3′; and control C2 (23). siRNAs were purchased from Dharmacon. HeLa and HEK-293T cells were typically maintained in 10 cm plates in dulbecco's modified Eagle's medium (DMEM) with glutamax (Gibco, Life technologies) supplemented with 10% (v/v) FCS. For splicing reporter assays, 3 × 10^5^ HEK-293T cells were plated in 35 mm diameter wells 24 h prior to transfection in 1.5 ml DMEM. A total of 200 ng splicing reporter and 400 or 800 ng GFP expression vector, made up to a total of 1 μg plasmid with pGEM4Z was transfected using 2 μl LipofectAMINE 2000™ reagent (Invitrogen). RNA and protein were harvested 48 h following transfection. For fluorescence microscopy experiments, HeLa cells grown on glass coverslips were transfected with 2 μg PTBP3-AUG1, PTBP3-AUG4, PTBP3-AUG11 pEGFP-N1 constructs using 4 μl LipofectAMINE 2000™ reagent (Invitrogen).

Primary B cell single cell suspensions were prepared from spleens of C57BL/6 mice by passing the spleen consecutively through a 70 and 40 μm nylon mesh. B cells were isolated by negative MACS sorting with the mouse B cell isolation kit from Miltenyi Biotec. B cells were cultured in IMDM media (Life Technologies) supplemented with 10% FCS (GIBCO), 50 μM β-mercaptoethanol (Sigma) penicillin and streptomycin (GIBCO) and stimulated with 10μg/ml LPS (127:B0, Sigma) for 48 h.

### Fluorescence microscopy

Twenty-four hours after transfection, cells were fixed in formalin (SIGMA), permeabilized in 0.5% Triton in phosphate buffered saline (PBS) for 5 min, blocked in 1% bovine serum albumin (BSA) in PBS, stained with anti-PTBP1 monoclonal antibody in PBS (1:200) for 1 h, washed with PBS and incubated with secondary mouse antibody conjugated to rhodamine (1:200) (Jackson ImmunoResearch) for 1 h. All incubations were performed at room temperature in the absence of light. Coverslips were mounted in Mowoil (Calbiochem). Cells were observed under an Olympus BX61 microscope with a 100X/1.30 oil immersion objective.

### Protein and RNA analysis

Total protein was harvested with RIPA buffer supplemented with protease inhibitors or by direct lysis in sodium dodecyl sulphate (SDS) loading buffer when total cell lysates were analysed. For cytoplasmic and nuclear fractions of cell lines 10^6^ cells were resuspend in 50 μl ice-cold buffer A (10 mM HEPES pH 7.9, 1.5 mM MgCl_2_, 10 mM KCl, 0.5 mM dithiothreitol (DTT), 0.2 mM phenylmethanesulfonylfluoride (PMSF)), incubated on ice for 30 min and lyzed with a pestle. Samples were centrifuged for 1 min at 13 000 r.p.m. Supernatant was collected as cytoplasmic fraction, nuclear pellets were resuspended in 50 μl of SDS loading buffer. Cytoplasmic and nuclear fractions of mouse B cells were harvested with cytosolic lysis buffer (10mM Tris–HCl, pH 7.4, 10mM NaCl, 2.5 mM MgCl_2_, 40 μg/ml digitonin and protease inhibitors) and total lysis buffer (50 mM Tris–HCl, pH 7.4, 100 mM NaCl, 1% NP-40, 0.1% SDS, 0.5% sodium deoxicholate and protease inhibitors). Equivalent amounts were separated in 12.5% (w/v) sodium dodecylsulphate-polyacrylamide gel electrophoresis (SDS-PAGE) except gels containing mouse B cell protein extracts which were separated in NuPAGE Novex 4–12% Bis-Tris Gels (Life Technologies).

Anti-PTBP1 mouse monoclonal antibody (BB7) was obtained from D. Black, anti-PTBP1 mouse monoclonal antibody (Clone 1) from Invitrogen, anti-PTBP2 rabbit polyclonal antibody from A. Willis, anti-ERK antibody from Santa Cruz and anti-GFP antibody from Molecular Probes/Invitrogen. Anti-Lamin A/C (636) and anti-Lamin A/C (H110) were obtained from Santa Cruz, anti-tubulin (YL1/2) was obtained from Abcam and anti-GAPDH (D16H11) was purchased from Cell Signalling.

Three anti-PTBP3 antibodies were used: rabbit polyclonal PTBP3-FL antibody, rabbit polyclonal PTBP3-L2–3 and rat anti-PTBP3 monoclonal antibody (MAC454). The rabbit polyclonal PTBP3-FL antibody was raised in a rabbit against PTBP3-AUG4 (Eurogentec) and affinity purified and depleted of PTBP1 and PTBP2 crossreacting antibodies as previously carried out for anti-PTBP2 (17). Rabbit polyclonal PTBP3-L2–3 antibody was raised in a rabbit (Cambridge Research Biochemicals) against recombinant GST-mouse Ptbp3 protein containing part the RRM2 to RMM3 linker (amino acids 279–359) of the AUG1 mouse Ptbp3 isoform. The antibody was first positively purified by affinity chromatography with the GST-mouse Ptbp3 recombinant protein used in the immunization and subsequently depleted of anti-GST and PTBP1 antibodies by negative affinity chromatography purification using a GST fusion recombinant protein containing part of the RRM2 to RRM3 linker of mouse Ptbp1 (278–360 amino acids from the mouse Ptb4 isoform). The rat monoclonal Ab MAC454 will be described in detail elsewhere. The antigen used for rat immunizations was the GST-mouse Ptbp3 RMM2 to RRM3 linker recombinant protein used also for the generation of the rabbit polyclonal PTBP3-linker-2–3 antibody. Where necessary, western blots were quantitated by densitometry of autoradiographs using Total lab TL120 software (Nonlinear Dynamics).

RNA was harvested with TRI reagent (Sigma) according to the manufacturer's protocol. Reverse transcription (RT) reactions were carried out using Superscript II (Invitrogen), 1–2 μg total RNA and oligo dT. Detection of PTBP3 transcripts by polymerase chain reaction (PCR) amplification used 1/20th of the RT reaction as template and 400 ng PTBP3–1aF primer (5′ TCCATCTGGGCCATGGAT 3′) and 160 ng ^32^P-5′-labelled PTBP3_5R primer (5′ CAGCTTCCTCAGAAGCCATT 3′). Reactions were separated by denaturing polyacrylamide gel electrophoresis and quantitated by phosphorimager. Analysis of the minigene reporters were carried out as described in (6,20). Analysis of *FAS* *in* *vitro* splicing was carried out as described in (24,25). Statistical significance between percent exon inclusion was assessed by unpaired two-tailed Student's *t*-test.

### Visualization of K562 and mouse B-cell RNAseq data

Two RNAseq libraries (GSM1289404 and GSM1289405) from K562 cells (26) were downloaded from the Gene Expression Omnibus (GEO) repository. Details for RNA purification, library preparation and sequencing can be found under the GEO accession numbers. RNAseq libraries were trimmed with Trim Galore (version 0.3.7, http://www.bioinformatics.babraham.ac.uk/projects/trim_galore/) using paired-end trimming mode and default parameters. Reads were mapped to the human hg19 genome. Mapping was carried out with TopHat version 2.0.12 (27) using -p 6 -g 1 parameters and the Homo_sapiens.GRCh37.61.gtf annotation. RNAseq libraries from mouse primary B cells (GSM1520107, GSM1520108, GSM1520109 and GSM1520110) have been described elsewhere (28). Reads were trimmed with TrimGalore (v0.3.3, default parameters). Mapping to the mouse mm10 genome was carried out with TopHat (v2.0.7) using -p 6 -g 1 parameters and the Mus_musculus.GRCm38.70.gtf annotation. Mapped reads were visualized in Sashimi plots generated with IGV (29). The minimum junction coverage was set to 30 for K562 cells and to 5 for mouse B cells when Sashimi plots were generated.

### Expression and purification of recombinant PTBP3 proteins

His-tagged PTBP3 proteins were expressed in *Escherichia coli* BL21 cells. Cells were lysed in 300 mM NaCl, 50 mM NaH_2_PO_4_ buffer (pH 8.0) supplemented with protease inhibitor cocktail (Roche) and His-tagged proteins were purified using Ni-NTA beads (QIAGEN). The purified proteins were eluted with 300 mM NaCl, 50 mM NaH_2_PO_4_ buffer (pH 8.0), 400 mM imidazole, supplemented with protease inhibitor cocktail (Roche), then dialyzed against 20 mM HEPES, pH 7.9, 300 mM KCl, 0.2 mM ethylenediaminetetraacetic acid, 0.5 mM DTT, 20% glycerol. The purified proteins were at least 90% pure, as verified by Coomassie staining of samples run on SDS-PAGE.

### RNA binding assays

RNAs were transcribed with T7 RNA polymerase (prepared in house) from a linearized DNA template incorporating trace amounts of [α^32^P]UTP. RNAs were phenol extracted, purified using a G50 Micro Column (ProbeQuant) and quantified by scintillation counter (Beckman LS 3801). For UV-crosslinking 5–10 fmol of RNA was incubated with 400 nM PTBP3 protein and 0.25 μg rRNA in 10 mM HEPES, pH 7.9, 3 mM MgCl_2_, 5% (v/v) glycerol, 1 mM DTT, 100 mM KCl at 30°C for 30 min. Heparin was added (0.33 mg/ml) 5 min before the end of the reaction. Samples were exposed to 2 × 960 mJ UVC per cm^2^ in a crosslinker (Spectronics) before digestion with RNase T1 (0.8 U/μl) and RNase A (0.28 mg/ml) (Sigma). Crosslinked proteins were resolved by SDS-PAGE and autoradiography.

For filter binding, 20 μl reactions were assembled in 96-well plates. Reactions contained 4 μl 5× binding buffer (50 mM HEPES, 15 mM MgCl2, 5 mM DTT, 25% (v/v) glycerol), 2 μl 0.5 mg/ml tRNA (Sigma), 0.8 μl 33U/μl RiboShield™ Ribonuclease inhibitor (Dundee Cell Products), 1 μl 100 μM BSA. To this 4 μl recombinant protein/buffer E and the appropriate dilution of RNA was added and final KCl adjusted to 100 mM. Reactions were incubated at 30°C, 15 min before being added to an assembled Whatman^®^ Minifold^®^ I 96 well dot-blot assembly with a Protran NBA-085B Nitrocellulose membrane (Whatman), and a Hybond-N (GE Healthcare). Both membranes were pre-incubated in wash buffer (10 mM HEPES, 2.5 mM MgCl_2_, 5% (v/v) glycerol, 1 mM DTT) for 1 h. Wells were first washed with 200 μl wash buffer, binding reactions were applied followed by 200 μl of wash buffer. Membranes were dried, exposed to a Phosphorimager screen (GE Healthcare) and scanned using a Storm Phosphorimager (GE Healthcare). Data was analysed using ImageQuant TL software (GE Healthcare). Dissociation constants were calculated as: *K*_D_ = [R][P]/[R.P] where [R], [P] and [PR] correspond to free RNA, free protein and bound complex concentrations respectively. All RNA concentrations were at least 100-fold lower than the lowest protein concentration, so total protein could be used as the free protein concentration. By plotting bound/free RNA versus the input protein concentration the gradient is equal to 1/*K*_D_. Statistical significance between *K*_D_s was assessed by unpaired two-tailed Student's *t*-test.

### Chymotryptic digestion

Reactions were performed in buffer E with 10 mM CaCl_2_. Recombinant protein and chymotrypsin (Sigma) were added to final concentration of 12.5 ng/μl and 0.21 ng/μl respectively. The reaction was incubated at 25°C and 10 μl fractions removed at set time points were added directly to 10 μl 2× SDS loading buffer at 90°C and incubated at 90°C for 2 min. The 0 min time point was removed prior to the addition of chymotrypsin and added directly to 10 μl 2× SDS loading buffer at 90°C, after which the chymotrypsin was added. A negative control sample was generated by removing a fraction prior to the addition of chymotrypsin and chymotrypsin buffer (1 mM HCl, 2 mM CaCl_2_) added instead of chymotrypsin. This sample was incubated at 25°C for the same length of time at the longest time point. All fractions were subjected to SDS-PAGE and visualized by silver staining.

## RESULTS

### Extensive alternative splicing of PTBP3

In addition to the skipping of *PTBP3* exon 2, exon 4 can be skipped and exon 3 has alternative 5′ splice sites (5′ ss) separated by 9 nt (Figure 1A). To determine the extent to which these splicing events occur in haematopoietic cells, we used primers in exons 1 and 5 to carry out RT-PCR analysis of transcripts from the human erythroleukaemia cell line, K562. Expression of 8 mRNA species was detected representing every combination of the annotated alternative splicing events (Figure 1B, lane 1). The alternative 5′ ss choice in exon 3 alters the transcripts by only 9 nt, whereas inclusion or skipping of exons 2 (34 nt) and/or 4 (170 nt) shifts and prematurely terminates the reading frame initiating at AUG1 in exon 1. We therefore focused on differences in splicing of exon 2 and exon 4 (Figure 1B). Full length *PTBP3* mRNA only comprises 10% of spliced transcripts, while transcripts with exon 2 skipped and exon 4 included were the most abundant at 48%. Transcripts with exon 4 skipped alone or in combination with exon 2 skipping comprised 13 and 29% of total mRNAs respectively. Analysis of available K562 cell mRNA-Seq datasets agreed with the RT-PCR data. Reads across exon-exon junctions show that most PTBP3 transcripts exclude exon 2, that there is roughly two-fold more use of the 3L 5′ ss of exon 3 than the 3s site, and that exon 4 is ∼80% included (Figure 1C). Moreover the RNAseq data shows no further major alternative splicing events downstream of exon 5. Despite the complexity of expressed mRNA isoforms, we detected only two major protein isoforms of ∼48 and 57 kDa (Figure 1E, lane 4), similar to earlier reports (19).

To examine the extent of crossregulation of *PTBP3* splicing, we looked for changes in splicing upon PTBP1/PTBP2 knockdown (Figure 1B, D and E). Upon knockdown of PTBP1, we observed upregulation of PTBP2 (Figure 1E, lanes 4,5), demonstrating that the PTBP1 crossregulation of PTBP2 also occurs in K562 cells. Indeed, analysis of *PTBP2* RNA showed an increase in exon 10 inclusion from 30 to 90% upon PTBP1 knockdown (Supplementary Figure S2). Upon combined PTBP1/PTBP2 knockdown, inclusion of *PTBP3* exon 2 increased from 23 to 84%. In contrast, splicing of exon 4 and use of the downstream 5′ ss in exon 3 did not change significantly, remaining around 60 and 64% respectively (Figure 1B and D). Therefore PTBP1 and PTBP2 regulate splicing of exon 2, but not the other splicing events in *PTBP3*. Western blot analysis showed the appearance of a larger PTBP3 protein isoform of ∼60 kDa upon PTBP1/PTBP2 knockdown (Figure 1E, lanes 5,8). This band, which was undetectable under control conditions (lane 4) represented ∼1% of total PTBP3 upon PTBP1 knockdown (lane 5) and 16% upon PTBP1/PTBP2 knockdown (lane 8). Knockdown of PTBP3 alone or in combination with PTBP1 and/or PTBP2 confirmed that the 48, 57 and 60 kDa isoforms are PTBP3 (Figure 1E, lanes 6, 7, 9). The size of the 60 kDa isoform is consistent with translation of the exon 2 included *PTBP3* mRNA using AUG1 as the initiating codon. Knockdown of PTBP3 had no effect upon expression of either PTBP1 or PTBP2 (Figure 1E lanes 6, 7; note that the apparent increase in PTBP1 levels in lane 6 was not observed in other experiments).

Skipping of exon 2 (34 nt) and/or exon 4 (170 nt) results in frameshift and introduction of a premature termination codon in the reading frame initiating at AUG1. However, exon 2 and exon 4 skipped transcripts were not stabilized upon UPF1 knockdown indicating that they are not NMD-sensitive (Supplementary Figure S3), in contrast to the regulated splicing events in *PTBP1* and *PTBP2* (15–17,22).

### PTBP3 protein isoforms arise from initiation at alternative AUGs

The preceding results raise questions about the relationship between the observed PTBP3 mRNA and protein isoforms, as well as the nature of the crossregulatory network between PTBP1, PTBP2 and PTBP3. The predicted translation products from the differentially spliced PTBP3 mRNA transcripts are shown in Figure 2. ORFs that can potentially produce PTBP3 proteins are shown in black and uORFs in grey. Only the longest mRNA isoform containing exon 2 and 4 has a PTBP3 encoding ORF commencing at AUG1 in exon 1. For all other isoforms, AUG1 initiates a uORF and potential PTBP3 encoding ORFs initiate at AUGs 4, 10, 11 and 12. Note that the AUGs are numbered according to their location in the longest mRNA isoform, although AUGs 6–10 are in exon 4 and so are missing in exon 4 skipped mRNA isoforms. AUG1 is in a good Kozak consensus (Table 1), is present in all mRNA isoforms and would be expected to be recognized by a scanning 43S initiation complex. Initiation at the downstream AUGs could occur by leaky scanning or translational re-initiation (30,31). Alternative 5′ ss choice at exon 3, alters the coding sequence by 9 nt, preserving the reading frame and inserting a GVY tripeptide into PTBP3 isoforms initiating at AUG1 or 4. However, when exon 4 is skipped, AUG5, which lies between the two 5′ ss of exon 3, could potentially initiate a PTBP3 encoding ORF (Figure 2C and D). To ensure that we examined all possibilities of AUG usage, our subsequent expression constructs were all based on use of the downstream 5′ ss in exon 3.

**Table 1.** Kozak sequence contexts of PTBP3 AUGs 1–12, classed as strong moderate or weak on the basis of the −3 (A > G) and +4 (G) positions

AUG	Translation context	Strength
Consensus	gcc**[a>g]**ccAUG**g**au	consensus
AUG1	ugg**g**ccAUG**g**au	strong
AUG2	ccauggAUG**g**ug	moderate
AUG3	gaucugAUGagc	weak
AUG4	uuu**a**ccAUGaau	strong
AUG5	guguguAUG**g**cu	moderate
AUG6	cag/augcuaAUG**g**ga	moderate
AUG7	aug**g**gaAUGaca	moderate
AUG8	aauuccAUGuga	weak
AUG9	cau**g**ugAUGuca	moderate
AUG10	cuuuucAUGuug	weak
AUG11	uua**g**aaAUG**g**cu	strong
AUG12	guu**a**cuAUG**g**ug	strong

**Figure 3.**
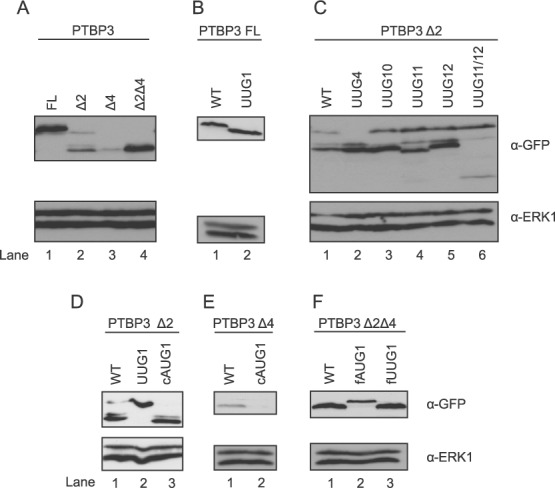
Translation initiation by PTBP3 proteins. Plasmids encoding PTBP3 fused to GFP at the C-terminal end were transfected into HEK293 cells and expression detected by western blot with anti-GFP antibodies (upper panels) and anti-ERK as a loading control (lower panels). UUG4, UUG10 etc. denotes AUG to UUG mutations at the indicated AUG. cAUG1 denotes a mutant creating a Kozak consensus around AUG1. fAUG1/fUUG1 denotes a UGA to UGC mutation in the stop codon that usually terminates the AUG1 uORF. (**A**) Wild-type PTBP3 isoforms. (**B**) Lane 1, WT FL PTBP3 and lane 2, AUG1/UUG1 mutant. (**C**) PTBP3 Δ2 mRNAs. Lane 1, WT. Lanes 2–6 AUG to UUG mutants. (**D**) PTBP3 Δ2 mRNAs. Lane 1, WT, Lane 2, UUG1, Lane 3, Kozak consensus AUG1. (**E**) PTBP3 Δ4. Lane 1, WT, lane 2, Kozak consensus AUG1. (**F**) PTBP3 Δ2Δ4. Lane 1, WT; lane 2, mutation of stop codon of AUG1 uORF; lane 3, AUG1 to UUG1 combined with mutation of stop codon of AUG1 uORF.

To determine which proteins are expressed from the different *PTBP3* transcripts, their cDNAs were cloned into mammalian expression vectors to express PTBP3-GFP fusion proteins. A total of four protein isoforms were detected (Figure 3A). The difference in size suggests that the three major isoforms correspond to the 48, 57 and 60 kDa isoforms observed in K562 and HeLa cells (Figure 1F and Supplementary Figure S4). To address whether these protein isoforms arise via alternative translation initiation we tested the effects of mutating the different potential initiating AUG codons.

The major protein produced by the full length PTBP3 construct had a size consistent with initiation at AUG1, with a minor product corresponding to initiation at AUG4 (Figure 3A, lane 1). Consistent with this, mutation of AUG1 to UUG1 resulted in complete loss of the larger band and exclusive production of a protein corresponding in size to the previously minor species (Figure 3B). These observations agree with the predictions that full length 60 kDa PTBP3 protein is produced from AUG1 in the full length mRNA (Figure 3A). Interestingly, the exon 2 skipped construct produced three PTBP3 proteins, the smallest of which was the major product (Figure 3A, lane 2, 3C, lane 1, PTBP3Δ2). A series of AUG to UUG mutations pinpointed the initiation codons associated with each of the three proteins (Figure 3C). The UUG4 mutation abolished the largest band and led to increased amounts of both smaller bands (Figure 3C, lane 2). This indicates that the largest band arises from initiation at AUG4, consistent with its comigration with the minor AUG4 product from FL PTBP3 (Figure 3A, lanes 1,2). The minor, intermediate sized band was abolished by the UUG10 mutation (Figure 3C lane 3) while the most abundant and most rapidly migrating band was abolished by the UUG11 mutation (Figure 3C lane 4). Concomitant with disappearance of the AUG11 band, a new slightly smaller band appeared in the UUG11 mutant. This was shown to arise from initiation at AUG12 by a double UUG11/UUG12 mutant in which both the AUG11 and AUG12 bands disappeared (Figure 3C lane 6). In contrast, the UUG12 mutation alone had no effect (Figure 3C, lane 5). These data demonstrate that the most abundant PTBP3 mRNA isoform in K562 cells (PTBP3Δ2) is able to produce the two major 57 and ∼48 kDa isoforms, as well as a third minor isoform. The only protein isoform that it is unable to generate is the 60 kDa protein initiating at AUG1. Moreover, these data confirm that the PTBP3 isoforms arise from translation initiation at alternative AUGs rather than from protein cleavage.

Further mutations were used to examine the possible translation initiation mechanism in the exon 2 skipped construct. Strikingly, AUG1 to UUG1 mutation in the PTBP3Δ2 construct abolished the AUG10 and AUG11 proteins, with the major product now initiating at AUG4 (Figure 3D lanes 1,2, Figure 2B). In contrast placing AUG1 in a perfect Kozak consensus (cAUG1, ugggccAUGga to gccaccAUGga) eliminated expression of the AUG4 isoform while leaving expression of the AUG11 isoform intact (Figures 3D, lane 3 and 2B). Taken together the data in Figure 3D indicates that the AUG10 and 11 products arise from re-initiation after translation of the AUG1 uORF while the AUG4 isoform arises from leaky scanning past AUG1 (summarized in Figure 2A).

The exon 4 skipped isoform showed very low levels of expression, with exclusive production of the AUG11 protein isoform (Figure 3A, PTBP3Δ4). The long AUG1 uORF in PTBP3Δ4 overlaps with AUG11 (Figure 2C), excluding the possibility of re-initiation after the AUG1 uORF (31). Mutation of AUG1 to a perfect Kozak consensus eliminated AUG11 expression, consistent with a leaky scanning mechanism to locate AUG11 in PTBP3Δ4 (Figure 3E, lane 2). In contrast, the double exon 2, exon 4 skipped PTBP3Δ2Δ4 construct showed efficient and almost exclusive expression of the AUG11 protein (Figure 3A, lane 4). The combined skipping of exon 2 and exon 4 puts AUG1 in the same reading frame with the remainder of PTBP3. Consistent with the idea that AUG11 use results from reinitiation after the AUG1 uORF, mutation of the stop codon of the AUG1 uORF allowed expression exclusively of PTBP3 protein from AUG1 (Figure 3F, fAUG1). This was confirmed by a double mutations in the start and stop codons of the AUG1 uORF, which produced exclusively the AUG11 isoform (Figure 3F, fUUG1).

Taken together, the data of Figure 3 show that the major PTBP3 isoforms in K562 cells arise from translation of mRNAs with exon 2 skipping, with initiation at AUG4 and 11, and minor amounts of an AUG10 isoform. Only the FL mRNA isoform with exons 2 and 4 included gives rise to a 60 kDa PTBP3 protein initiating at AUG1. The use of these AUGs and no others is consistent with their Kozak sequence contexts (Table 1). The mutational analysis here indicates that re-initiation and leaky scanning are the major mechanisms that allow expression of the smaller AUG11 and AUG4 PTBP3 isoforms in the exon 2 and 4 skipped mRNAs.

Having characterized the nature of the PTBP3 isoforms, we were next interested in the possible functional differences. The AUG1 isoform aligns well throughout its length with PTBP1 and PTBP2 (Supplementary Figure S1), while initiation at AUG4 and 11 causes N-terminal truncations that might be expected to alter some functions (Figure 2A). The AUG4 isoform lacks 31 N-terminal residues including part of the bipartite NLS (K_14_R_15_) (32,33), as well serine-17, which in PTBP1 is phosphorylated by protein kinase A shifting localization to the cytoplasm (12). Initiation at AUG11 has even more dramatic consequences, deleting 103 N-terminal amino acids including the entire N-terminal leader and most of RRM1. Only α helix 2 and β strand 4 of RRM1 remain, so this isoform might be expected to show differences in RNA binding as well as localization.

### RNA binding by PTBP3 isoforms

**Figure 4.**
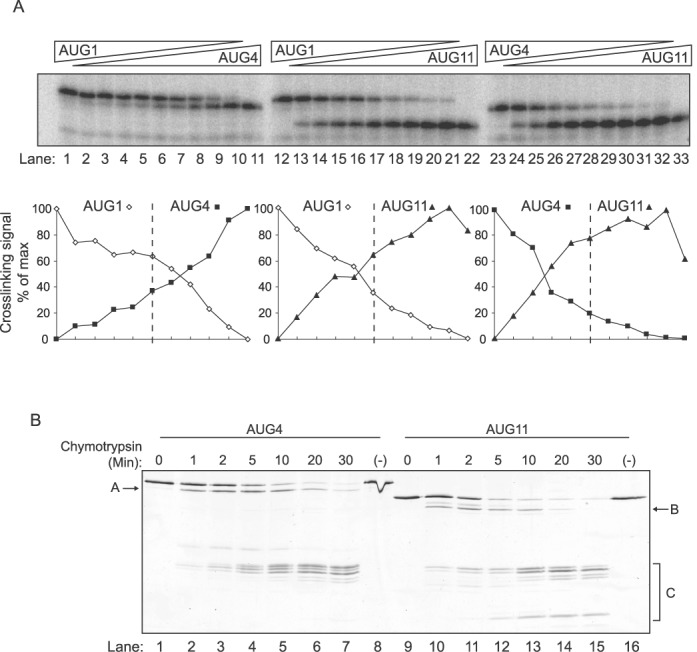
RNA binding and chymoytryptic sensitivity of PTBP3 isoforms. (**A**) PTBP3 proteins were incubated with radiolabelled MTDH exon 7 RNA probe. Total PTBP3 protein concentration was 400 nM. Lanes 1, 12: AUG1. Lanes 11, 23: AUG4. Lanes 22, 33: AUG11. Lanes 1–11, 12–22 and 23–34 show cross-tirations of AUG1 versus AUG4, AUG1 versus AUG11 and AUG4 versus AUG11. Nanomolar ratios are: 400:0, 380:20, 350:50, 300:100, 250:150, 200:200, 150:250, 100:300, 50:350, 20:380, 0:400. The graphs below show the percentage maximal crosslinking for each individual protein. The vertical dashed line indicates the equimolar 200:200 ratio, which should be the crossover point if the two isoforms bind with identical affinity. (**B**) Limited chymotrypsin digestion of PTBP3 AUG4 (lanes 1–8) and AUG11 (lanes 9–16). Band labelled ‘A’ corresponds to cleavage at Y42/F51 in the N-terminal leader. Band ‘B’ corresponds to cleavage within the part of RRM1 remaining in AUG11 at Y116, Y117, Y130 or Y133. Bands bracketed as ‘C’ correspond to multiple cleavages in the linker between RRMs 2 and 3.

To investigate possible differences in RNA binding of the PTBP3 isoforms we prepared recombinant proteins by expression in *E. coli*. RNA binding by all three PTBP3 isoforms was readily detected by UV-crosslinking to a probe from MTDH exon 7 (Figure 4A, lanes 1, 11, 12, 22, 23, 33)(6). The intensity of UV cross-linking by the AUG11 isoform was greater than that of AUG1 and 4. However, this could be related to differential efficiency of photochemistry rather than real differences in binding. To obtain a semi-quantitative comparison of binding by the three PTBP3 isoforms, we carried out competitive UV-crosslinking with cross-titrations of each pairwise combination of PTBP3 isoforms, maintaining a constant total concentration of PTBP3 (Figure 4A). The relative crosslinking signal was normalized to the maximum signal for each isoform, to account for any isoform-specific variation in crosslinking efficiency. At the midpoint of the cross-titration the concentration of each isoform is equal (dotted line). Therefore if the crosslinking signal favours one of the isoforms then that protein has higher affinity for the RNA. The most striking observation was that the AUG11 isoform, which lacks most of RRM1, showed no deficiency in crosslinking. Indeed AUG11 crosslinked with higher efficiency than AUG4 and with equal efficiency to the AUG1 isoform.

To compare binding affinities directly we used filter binding assays with a range of RNA substrates including unstructured (MTDH exon 7 and [CUCUCU]_4_) and structured PTB-binding RNAs (Poliovirus IRES and EMCV IRES). For these assays we directly compared PTBP3 AUG4 and AUG11 isoforms; we were unable to obtain sufficient quantities of purified AUG1 protein for the full range of comparisons. We also tested an artificial PTBP3 deletion mutant completely lacking RRM1. In agreement with the UV competition cross-linking (Figure 4A), the AUG11 isoform had a significantly lower *K*_D_ than AUG4 for binding to MTDH exon 7 and to PV IRES (Table 2). For the other two RNAs the dissociation constants for the two isoforms were not significantly different. In comparison with the ΔRRM1 protein, AUG11 had significantly lower *K*_D_ for binding to MTDH exon 7 and [CUCUCU]_4_, suggesting that the residual part of RRM1 plays a role in RNA binding. The affinities of the ΔRRM1 and AUG11 proteins for the PV IRES were similar, and both had significantly lower *K*_D_s than AUG4. Therefore, for some RNAs, as exemplified by PV IRES, a partial or complete lack of RRM1 leads to higher affinity binding. In contrast, for MTDH exon 7, the AUG11 isoform binds with higher affinity than either AUG4 (with intact RRM1) or ΔRRM1.

**Table 2.** Dissociation constants for binding of PTBP3 proteins to different RNAs

A			
RNA	AUG4	AUG11	ΔRRM1
MTDH exon 7	91.5 ± 6.6 nM	63.2 ± 3.8 nM	91.1 ± 4.7 nM
[CUCUCU]_4_	34.8 ± 7.8 nM	43.7 ± 4.8 nM	75.8 ± 11.9 nM
PV IRES	44.1 ± 2.0 nM	23.4 ± 5.5 nM	23.6 ± 4.7 nM
EMCV IRES	6.4 ± 1.3 nM	4.3 ± 0.6 nM	6.5 ± 1.1 nM

B			
RNA	AUG4 versus AUG11	AUG11 versus ΔRRM1	AUG4 versus ΔRRM1
MTDH exon 7	0.003	0.001	0.932
[CUCUCU]_4_	0.169	0.012	0.008
PV IRES	0.004	0.955	0.002
EMCV IRES	0.083	0.051	0.965

**A**; Average Kd ± standard deviation. **B**; Statistical significance of difference in binding between pairs of PTBP3 proteins. *P*-values calculated using a Student's *t*-test.

The higher affinity of AUG11 for some RNAs compared to either AUG4 or ΔRRM1, suggested that the residual part of RRM1 might be able to fold in some way so as to promote RNA binding. As an initial step to probe the structure of the AUG11 protein we carried out limited chymotryptic digestion (Figure 4B). Chymotrypsin cleaves adjacent to the aromatic side-chains of tryptophan, phenylalanine and tyrosine. In natively folded globular domains, most of these residues are buried and inaccessible. However, residues in unstructured regions will be accessible for recognition by chymotrypsin. Consistent with this expectation limited treatment of PTBP3 AUG4 with chymotrypsin led to preferential cleavage in the N-terminal leader sequence (Y42/F51, band A) and in the linker between RRMs 2 and 3 (F298, Y308, F314, F320, bands C, Figure 4B, lanes 1–8). There was no evidence of cleavage within any of the RRM domains. The AUG 11 isoform also showed the cleavages between RRMs 2 and 3 (bands ‘C’, lanes 9–16). However, AUG11 was also cleaved at positions corresponding to Y116, Y117, Y130 and Y133 in the residual part of RRM1 (Figure 4B lanes 9–16, band ‘B’). These residues were not accessible in AUG4, where RRM1 is intact, as shown by the lack of band B in lanes 1–7. These data suggest that the residual part of RRM1 in the AUG11 isoform does not adopt a stable fold in which the tyrosine residues are inaccessible to chymotrypsin. We considered the possibility that AUG11 might undergo a conformational change upon RNA binding. However, addition of excess [CUCUU]_8_ RNA had no effect upon the chymotryptic sensitivity of AUG11 (Supplementary Figure S5).

### Localization and splicing activity of PTBP3 protein isoforms

**Figure 5.**
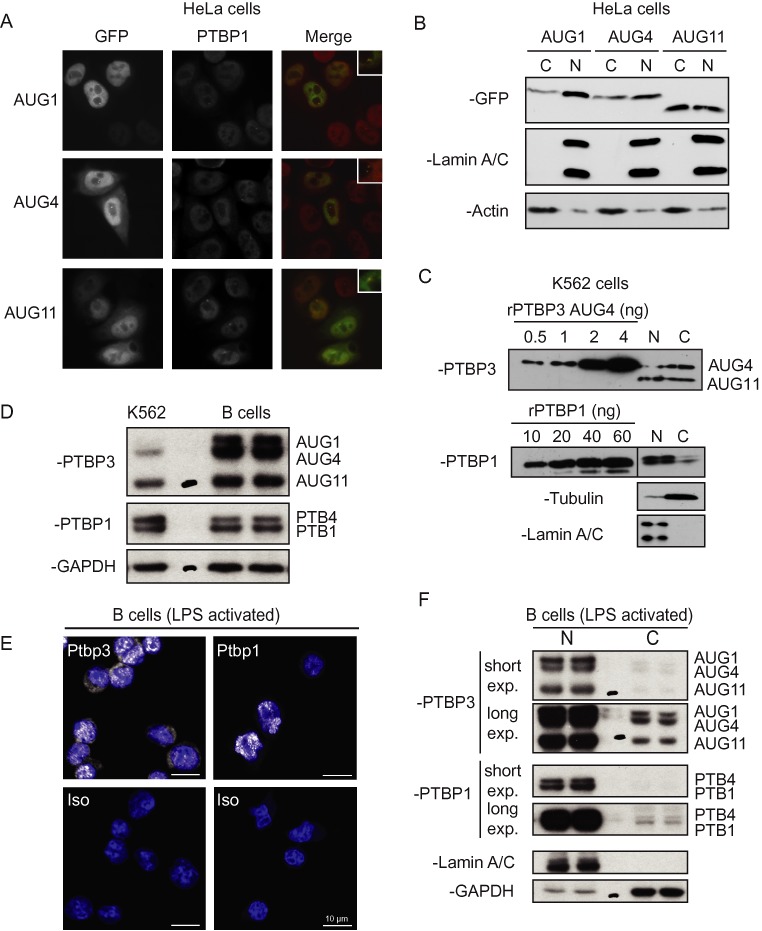
Localization of PTBP3 isoforms. Plasmids encoding PTBP3-GFP isoforms initiating at AUG1, 4 or 11 were transfected into HeLa cells and analysed by (**A**) fluorescence microscopy, or (**B**) by cellular fractionation and western blot. (A) Cells were imaged for GFP fluorescence, left column and by immunofluorescence with PTBP1 antibodies (BB7, middle column). Merged images are shown in the right column. (B) Western blots of cytoplasmic ‘C’ and nuclear ‘N’ fractions from cells transfected with the PTBP3 AUG1, 4 and 11 GFP fusions. Lamin A/C (626 antibody) is a nuclear marker, while α-actin is cytoplasmic. (**C**) Western blot of nuclear ‘N’ and cytoplasmic ‘C’ fractions from K562 cells, with Lamin A/C and Tubulin as nuclear and cytoplasmic markers. Antibodies used were α-PTBP1 (BB7) and α-PTBP3-FL. A titration of recombinant PTBP1 and PTBP3 (AUG4 isoform) was run on the same gel to allow estimation of levels of PTBP1 and PTBP3. (**D**) Western blots of total protein extracts derived from K562 and mouse B cells (duplicate samples). PTBP3 was detected with the α-PTBP3 MAC454 antibody and PTBP1 with Clone 1 monoclonal antibody. GAPDH is show as loading control. (**E**) Immunofluorescence microscopy of mouse primary B cells activated with LPS for 48 h. Ptbp3 and Ptbp1 (in white) were detected with α-PTBP3-L2–3 polyclonal and α-PTBP1 (Clone 1) monoclonal antibodies, respectively. Stainings of pictures at the bottom were carried out with the respective isotype-matched negative control antibodies. DNA staining (in blue) shows the nucleus. (**F**) Western blots of cytoplasmic (C) and nuclear (N) fractions from LPS (48 h) activated mouse B cells. PTBP1 and PTBP3 were detected as in D. Lamin A/C (H110 antibody) is a nuclear marker, while GAPDH is cytoplasmic.

The AUG4 and AUG11 PTBP3 isoforms both lack the first half of the bipartite NLS (Figure 2A, Supplementary Figure S1). To test whether this affects their localization we used fluorescence microscopy of HeLa cells transfected with expression constructs for GFP-fusions of the PTBP3 isoforms (Figure 5A, GFP) and compared this to endogenous PTBP1 by immunofluorescence (Figure 5A, PTBP1). Full length PTBP3-AUG1 was predominantly nuclear, similar to endogenous PTBP1. However, both PTBP3-AUG4 and PTBP3-AUG11 showed substantial accumulation in the cytoplasm as well as the nucleus (Figure 5A, middle and lower rows). Western blots of nuclear and cytoplasmic fractions also showed that full length PTBP3-AUG1 was predominantly nuclear, while PTBP3-AUG4 and PTBP3-AUG11 showed an even nuclear and cytoplasmic distribution (Figure 5B). The difference in localization between the isoforms is most likely due to the lack of a complete NLS in the AUG4 and 11 isoforms. We next analysed the nuclear/cytoplasmic distribution of PTBP1 and PTBP3 in K562 cells by western blot (Figure 5C). While PTBP1 showed the expected predominant nuclear localization, both the AUG4 and AUG11 isoforms of PTBP3 showed a more even distribution between nuclear and cytoplasmic fractions. Thus, the PTBP3 AUG4 and AUG11 isoforms endogenous to K562 cells show a similar cellular distribution to the transfected constructs in HeLa cells. To analyse the localization of PTBP3 and PTBP1 in a more physiological context, we turned to mouse B-cells (Figure 5D-F). Western blots indicated that B cells contained higher overall levels of PTBP3 than K562 cells (2.4–2.7-fold higher, normalized to GAPDH, Figure 5D). Moreover, while the AUG1 isoform was undetectable in K562 cells, it represented 24% of PTBP3 in B-cells (Figure 5D). Consistent with this, mRNA-Seq data showed that levels of both exon 2 and exon 4 inclusion are higher in B-cells than K562 (Supplementary Figure S6). In contrast, the levels of PTBP1 in B-cells were 70% of the levels in K562 (normalized to GAPDH). Immunofluorescence analysis of mouse B-cells indicated that PTBP1 was restricted to the nuclei of mouse B-cells, but PTBP3 was observed in both the nucleus and cytoplasm (Figure 5E). Examination of nuclear and cytoplasmic enriched fractions from mouse B-cells by western blots (Figure 5F) also indicated that a higher proportion of cytoplasmic PTBP3 than PTBP1. Moreover, the full length AUG1 isoform represented 36% of nuclear PTBP3, but 24% of the cytoplasmic fraction.

**Figure 6.**
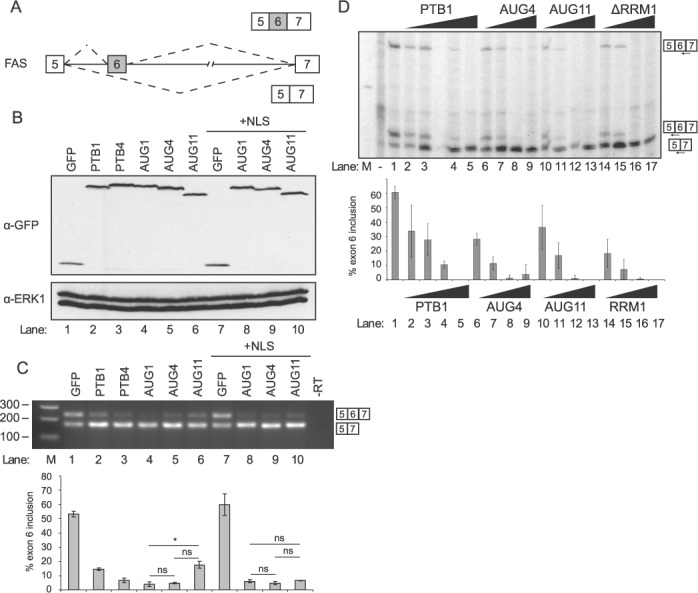
PTBP3 isoforms act as repressors of *FAS* exon 6. (**A**) Schematic of *FAS* minigene constructs used for transfection (panels **B** and **C**) and *in vitro* splicing (panel **D**). (B) Western blot of HEK239T cells transfected with the indicated GFP fusion proteins. Top panel: anti-GFP. Lower panel: anti-ERK. (C) RT-PCR analysis of *FAS* minigene splicing using primers in exon 5 and 7. The histogram below shows the percentage exon 6 inclusion (mean ± s.d., *n* = 3). D) *In vitro* splicing in HeLa nuclear extract of *FAS* RNA, with detection of products by primer extension. Lanes 2–5, 6–9, 10–13, 14–17, contain titrations (75, 150, 300 and 900 nM) of the indicated recombinant proteins: PTB1 isoform of PTBP1 (lanes 2–5), PTBP3 AUG4 (lanes 6–9), PTBP3 AUG11 (lanes 10–13), PTBP3 ΔRRM1 mutant (lanes 14–17). The histogram below shows the percentage of exon 6 spliced in (mean ± s.d., *n* = 3). All protein concentrations above 75 nM produced significantly increased exon skipping compared to nuclear extract alone (*P* < 0.01, Students two-tailed *t*-test).

We next compared the activities of the PTBP3-AUG1, -AUG4 and -AUG11 isoforms and the PTB1 and PTB4 isoforms of PTBP1, upon splicing of *FAS* exon 6 (Figure 6A) (20,24). Since it has been previously shown that the suboptimal codon content of PTBP3 limits expression in our assays, similar to PTBP2 (20), the GFP-PTBP3 constructs contained a codon optimized sequence for efficient mammalian expression. As a result, all the proteins were expressed to similar levels (Figure 6B). When transfected into HEK-293T cells the *FAS* reporter RNA produces ∼50% exon 6 inclusion (Figure 6C, lane 1). All of the PTBP1 and PTBP3 isoforms showed splicing repressor activity, increasing exon 6 skipping. However, the PTBP3-AUG11 isoform was slightly weaker than the AUG1 or AUG4 isoforms (Figure 6C, lanes 4–6). The lower activity of the AUG11 isoform could be due to reduced nuclear localization. Tagging the PTBP3 isoforms with an additional NLS to direct nuclear localization removed the differences in activity (Figure 6C, compare lanes 4–6 and 8–10), suggesting that in this assay the lower activity of the AUG11 isoform is primarily associated with its localization. The PTBP1 and PTBP3 isoforms showed a very similar trend in activity for the regulation of MTDH exon 7 (Supplementary Figure S7). To directly compare the splicing repressor activity of the PTBP3 isoforms with PTBP1 we carried out *in vitro* splicing of a *FAS* exon 5–6–7 construct in HeLa nuclear extract (Figure 6D) (24,25). Recombinant PTBP1 and PTBP3 isoforms were expressed and purified from *E coli*. *FAS* exon 6 was included to ∼60% in nuclear extract (Figure 6D, lane 1) but the level of exon skipping increased to ∼100% upon addition of PTBP1 (Figure 6D, lanes 2–5). The PTBP3 AUG4 and AUG11 isoforms showed identical repressor activity (lanes 6–13, *P* > 0.37 for all comparisons at the same concentration). We also tested an artificial PTBP3 mutant that completely lacks RRM1. This protein also had identical activity to the natural PTBP3 isoforms, showing that RRM1 is dispensable for regulation of *FAS* splicing. Taken together, these data suggest that the PTBP3 isoforms have inherently similar splicing activity upon *FAS* exon 6, but that their activity can be modulated by their nuclear/cytoplasmic distribution.

## DISCUSSION

Our data clarify the identity of the major PTBP3 protein isoforms and how they are generated by the variety of mRNA isoforms. The original report of PTBP3 (then named ROD1) identified the AUG4 isoform as the likely protein product of an mRNA with exon 2 skipped but exon 4 included, and noted that the N-terminus was truncated compared to PTBP1 (19). How AUG4 would be selected for translation initiation, despite the presence of AUG1 in a good Kozak consensus context, was not addressed. Western blots also identified a protein of 50 kDa, but its nature was unclear. Our data show that the 50 kDa protein arises from translation initiation at AUG11 to yield a protein with a truncated RRM1. Notably, although this is the most abundant PTBP3 isoform in K562 and HeLa cells (Figures 1E and 5D, Supplementary Figure S4) AUG11 has not been annotated as a start codon in any REFSEQ or ENSEMBL isoforms. We also show that a full length PTBP3 protein, corresponding throughout its length to isoform 4 of PTBP1 (Supplementary Figure S1) is the sole translational product of full length PTBP3 mRNA containing exons 2 and 4. This protein is not usually evident in cultured cells such as K562 or HeLa due to the repressive influence of PTBP1 upon PTBP3 exon 2 (Figure 1, Supplementary Figure S2)(17). However, in mouse B-cells, where PTBP1 levels were lower, the AUG1 PTBP3 was readily detectable (Figure 5). All of the PTBP3 mRNA isoforms contain AUG1 in exon 1. Selection of the AUG4 and AUG11 initiation codons therefore occurs by a mixture of mechanisms other than conventional cap-dependent scanning. Leaky scanning to allow use of AUG4 in FL and Δ2 PTBP3 and of AUG11 in the Δ4 PTBP3 mRNAs was demonstrated by suppression of these products when the AUG1 consensus was improved to a perfect Kozak context. Most of the AUG11 product is produced via reinitiation after translation of uORFs initiating at AUG1 as indicated by the deleterious effects of mutating AUG1. The influences of uORFs as regulators of gene expression (34) and of the short peptides encoded by them (35) are increasingly appreciated. The relative importance of the non-conventional mechanisms for translation of AUG4 and 11 PTBP3 isoforms for quantitative regulation of translation, compared to the intrinsic functional differences of the PTBP3 protein isoforms remains to be established.

We examined potential functional differences between the three PTBP3 isoforms, including RNA binding (Figure 4), localization (Figure 5) and splicing regulation of *FAS* exon 6 (Figure 6). The clearest observed difference was in the higher degree of cytoplasmic localization of AUG4 and 11 compared with AUG1 (Figure 5), consistent with truncation of the NLS (Supplementary Figure S1). Surprisingly, the truncation of RRM1 in the AUG11 PTBP3 did not impair binding to several RNA substrates and was actually associated with higher affinity binding to some RNAs (Figure 4A, Table 2), and unimpaired splicing regulatory activity (Figure 6, Supplementary Figure S7). The lack of effect on regulation of *FAS* exon 6 splicing was surprising given that point mutations that impaired RNA contact by RRM1 of PTBP1 led to a ∼50% loss of activity (25). The relatively mild consequences of RRM1 truncation in PTBP3 AUG11 contrast with the severely impaired RNA binding and dominant negative function of an isoform of RbFox2 with a truncation of its single RRM domain (36).

A clearer view of the functional differences between PTBP3 isoforms requires better knowledge of the cellular functions and molecular targets of PTBP3. Our analyses were based on the expectation, informed by the high level of sequence identity (Supplementary Figure S1) that PTBP3 will play similar roles to PTBP1 in regulation of splicing in the nucleus. The greater cytoplasmic accumulation of the AUG4 and AUG11 PTBP3 isoforms (Figure 5) suggest that it might have even more prominent cytoplasmic roles in mRNA stability and translation than PTBP1 (10). Moreover, by analogy with the unique non-redundant roles of PTBP2 in neurons (8,14,18), we expect that PTBP3 will also have unique specialized roles, presumably in cells of the haematopoietic system. In an initial screen for endogenous PTBP3 targets in K562 cells, we tested the response of 17 known PTBP1 targets (6) to knockdown of PTBP3, alone or in combination with PTBP1 and PTBP2 (Supplementary Figure S8, Supplementary Table S1). Only three events responded to PTBP3 knockdown, including MTDH exon 7 (Supplementary Figure S7), although in each case the magnitude of change was small. In a further unbiased attempt to identify PTBP3 functions in splicing and translation regulation in K562 cells, we analysed the global consequences of PTBP3 knockdown using splice sensitive HJAY microarrays (6) and the polyosome:monosome ratio of cytoplasmic mRNAs. However, we found no significant changes in transcript levels, alternative splicing or polysome loading (PW and CWJS, unpublished observation). PTBP3 was reported to influence NMD in HEK293 cells (21). However, we observed no changes in transcript levels that would be consistent with such a role in K562 cells. Our data therefore indicates that PTBP3 does not play an important non-redundant role in K562 cells, despite the similar levels of PTBP1 and PTBP3 mRNAs and the readily detectable PTBP3 expression (Figure 1, (19)). Consistent with this, western blots with reference recombinant PTBP1 and PTBP3 protein, indicated that PTBP1 is present at levels ∼20-fold higher than PTBP3 in K562 cells (Figure 5C)).

A recently published ‘draft map’ of the human proteome indicated that PTBP3 is most highly expressed in B-cells and T-cells (37), with relatively lower levels of PTBP1 and 2 in B-cells. In agreement with this, we observed higher levels of PTBP3 relative to PTBP1 in B-cells (Figure 5D). Moreover, the high levels of the full length AUG1 PTBP3 isoform (Figure 5D), indicate relaxation of the PTBP1 repression of full-length PTBP3 isoform expression. This suggests that PTBP3 plays important non-redundant roles in B-cells. We are currently in the process of generating mice in which PTBP3 can be conditionally ablated in lymphoid cells *in vivo*. Our data on the diversity of PTBP3 mRNA isoforms and the ways in which they can initiate translation has been informative in developing a strategy for design of a PTBP3 knockout that should lack the ability to generate any functional proteins. The phenotypes of these mice should indicate the cell-types and developmental stages at which PTBP3 plays key non-redundant roles. We will then be better placed to examine in detail the functional differences in the isoforms of PTBP3.

PTBP1 and 2 both promote skipping of PTBP3 exon 2 (Figure 1), but unlike the regulated splicing events in PTBP1 (22) and PTBP2 (15–17), this splicing event does not downregulate PTBP3 mRNA levels (Supplementary Figure S3). Translation initiation by leaky scanning and/or reinitiation is expected to be less efficient than scanning to the cap-proximal AUG (31). Nevertheless, it is notable that PTBP1/PTBP2 knockdown led to the appearance of the full length AUG1 PTBP3 isoform without a major increase in the quantity of PTBP3 protein (Figure 1). This suggests that other post-transcriptional mechanisms might operate to limit the expression of PTBP3 outside the appropriate cell type. These mechanisms might include the influence of micro-RNAs, the suboptimal codon content of PTBP3 (mammalian expression constructs used here were codon optimized, as had previously been carried out for PTBP2 (20)), and also its GC-rich 5′ UTR, which contains predicted G-quadruplex structures which can render translation particularly sensitive to the availability of helicases (38). Future investigations will focus on both the physiological roles of PTBP3 as well as the various ways in which its expression is regulated. Finally, although the PTBP1/PTBP2 regulated splicing of PTBP3 does not act as an ON/OFF switch, it will be interesting to investigate the extent to which this event is a target of other splicing regulators in lymphoid cell splicing regulatory networks (1,39). We anticipate that a change in the balance of PTBP3 versus PTBP1 activity will play an important role in lymphoid cell physiology perhaps analogous to the interplay between hnRNPL and hnRNPLL during T-cell activation (40–43).

## SUPPLEMENTARY DATA

Supplementary Data are available at NAR Online.

SUPPLEMENTARY DATA
