# Supplementary material for: Generation of functionally distinct isoforms of PTBP3 by alternative splicing and translation initiation
Source: Nucleic Acids Res. 2015 May 4;43(11):5586–600. doi: 10.1093/nar/gkv429 (PMC4477659; doi:10.1093/nar/gkv429)
Supplement: SUPPLEMENTARY DATA [file supp_43_11_5586__index.html]

Generation of functionally distinct isoforms of PTBP3 by alternative splicing and translation initiation — Generation of functionally distinct isoforms of PTBP3 by alternative splicing and translation initiation — SUPPLEMENTARY DATA 

# Generation of functionally distinct isoforms of PTBP3 by alternative splicing and translation initiation

## SUPPLEMENTARY DATA

**Files in this Data Supplement:**

- SUPPLEMENTARY DATA
