# Supplementary material for: Generation of functionally distinct isoforms of PTBP3 by alternative splicing and translation initiation
Source: Nucleic Acids Res. 2015 May 4;43(11):5586–600. doi: 10.1093/nar/gkv429 (PMC4477659; doi:10.1093/nar/gkv429)
Supplement: SUPPLEMENTARY DATA [file supp_gkv429_nar-00043-a-2015-File008.pdf]

```
                 AUG1                                   AUG4
PTBP3            MDGVVTDLITVGLKRGSDELLSSGIINGPFT-MNSSTPSTGVYANGNDSKKFKRD-RPPCS 59
PTBP1            MDGIVPD-IAVGTKRGSDELFSTCVTNGPFI-MSSNSASA---ANGNDSKKFKGDSRSAGV 56
PTBP2            MDGIVTE-VAVGVKRGSDELLSGSVLSSPNSNMSSMV----VTANGNDSKKFKGEDKMDGA 56
                 ***:*.: ::** *******:*  : ..*   *.*.       ********** : :
                                                                 AUG11
PTBP3            PSRVLHLRKIPCDVTEAEIISLGLPFGKVTNLLMLKGKSQAFLEMASEEAAVTMVNYYTP 119
PTBP1            PSRVIHIRKLPIDVTEGEVISLGLPFGKVTNLLMLKGKNQAFIEMNTEEANTMVNYYTS 116
PTBP2            PSRVLHIRKLPGEVTETEVIALGLPFGKVTNILMLKGKNQAFLELATEEAAITMVNYYSA 116
                 ****:*:**:* :*** *:*:**********:******.***:*: :**** ******:.

PTBP3            ITPHLRSQPVYIQYSNHRELKTDNLPNQARAQAALQAVSAVQSGSLALSG--GPSNEGTV 177
PTBP1            VTPVLRGQPIYIQFSNHKELKTDSSPNQARAQAALQAVNSVQSGNLALAASAAAVDAGMA 176
PTBP2            VTPHLRNQPIYIQYSNHKELKTDNTLNQ-RAQAVLQAVTAVQTANTPLSG--TTVSESAV 173
                 :** **.**:***:***:*****.  ** ****.****.:**:.. .*:.   . . . .

PTBP3            LPGQSPVLRIIIENLFYPVTLEVLHQIFSKFGTVLKIITFTKNNQFQALLQYADPVNAHY 237
PTBP1            MAGQSPVLRIIVENLFYPVTLDVLHQIFSKFGTVLKIITFTKNNQFQALLQYADPVSAQH 236
PTBP2            TPAQSPVLRIIIDNMYYPVTLDVLHQIFSKFGAVLKIITFTKNNQFQALLQYGDPVNAQQ 233
                  ..********::*::*****:**********:*******************.***.*:

PTBP3            AKMALDGQNIYNACCTLRIDFSKLTSLNVKYNNDKSRDFTRLDLPTGDGQPSLEPPMAAA 297
PTBP1            AKLSLDGQNIYNACCTLRIDFSKLTSLNVKYNNDKSRDYTRPDLPSGDSQPSLDQTMAAA 296
PTBP2            AKLALDGQNIYNACCTLRIDFSKLVNLNVKYNNDKSRDYTRPDLPSGDGQPALDPAIAAA 293
                 **::********************..************:** ***:**.**:*: .:***

PTBP3            FGAPGIIS-SPYAGAAGFAPAIGFPQATGLSVPAVPGALGPLTITSS------AVTGRMA 350
PTBP1            FGAPGIISASPYAG-AGFPPTFAIPQAAGLSVPNVHGALAPLAIPSAAAAA--AAAGRIA 353
PTBP2            F-------------------------AKETSLLAVPGALSPLAIPNAAAAAAAAAAGRVG 328
                 *                         *   *:  * ***.**:*..:      *.:**:.

PTBP3            IPGASGIPGNSVLLVTNLNPDLITPHGLFILFGVYGDVHRVKIMFNKKENALVQMADANQ 410
PTBP1            IPGLAG-AGNSVLLVSNLNPERVTPQSLFILFGVYGDVQRVKILFNKKENALVQMADGNQ 412
PTBP2            MPGVSA-GGNTVLLVSNLNEEMVTPQSLFTLFGVYGDVQRVKILYNKKDSALIQMADGNQ 387
                 :** :.  **:****:*** : :**:.** ********:****::***:.**:****.**

PTBP3            AQLAMNHLSGQRLYGKVLRATLSKHQAVQLPREGQEDQGLTKDFSNSPLHRFKKPGSKNF 470
PTBP1            AQLAMSHLNGHKLHGKPIRITLSKHQNVQLPREGQEDQGLTKDYGNSPLHRFKKPGSKNF 472
PTBP2            SQLAMNHLNGQKMYGKIIRVTLSKHQTVQLPREGLDDQGLTKDFGNSPLHRFKKPGSKNF 447
                 :****.**.*::::** :* ****** ******* :*******:.***************

PTBP3            QNIFPPSATLHLSNIPPSVTVDDLKNLFIEAGCSVKAFKFFQKDRKMALIQLGSVEEAIQ 530
PTBP1            QNIFPPSATLHLSNIPPSVSEEDLKVLFSSNGGVVKGFKFFQKDRKMALIQMGSVEEAVQ 532
PTBP2            QNIFPPSATLHLSNIPPSVAEEDLRTLFANTGGTVKAFKFFQ-DHKMALLQMATVEEAIQ 506
                 *******************: :**: **  . *  **.***** *:****:*:.:****:*

PTBP3            ALIELHNHDLGENHHLRVSFSKSTI 555
PTBP1            ALIDLHNHDLGENHHLRVSFSKSTI 557
PTBP2            ALIDLHNYNLGENHHLRVSFSKSTI 531
                 ***:***::****************
```

Supplementary Fig 1. ClustalW2 alignment of human PTBP3 (ROD1), PTBP1 and PTBP2 (nPTB). The PTB paralogs share the same domain organization and a high degree of sequence identity. The RRMs (blue text) and RRM3-4 interdomain linker are also highly conserved. Also indicated are the conserved bipartite NLS (red) with the overlapping PKA phosphorylation site (underlined), the NES (boxed). Secondary structural elements in RRMs are shaded grey (β-strands) and yellow (α helices). Internal methionines initiating the truncated PTBP3 isoforms identified here are shown in red, and indicated above by AUG4 and AUG11. Amino acids identical between all three proteins are indicated by "*", the conserved substitutions by ":" and the semi-conserved ones with ".".

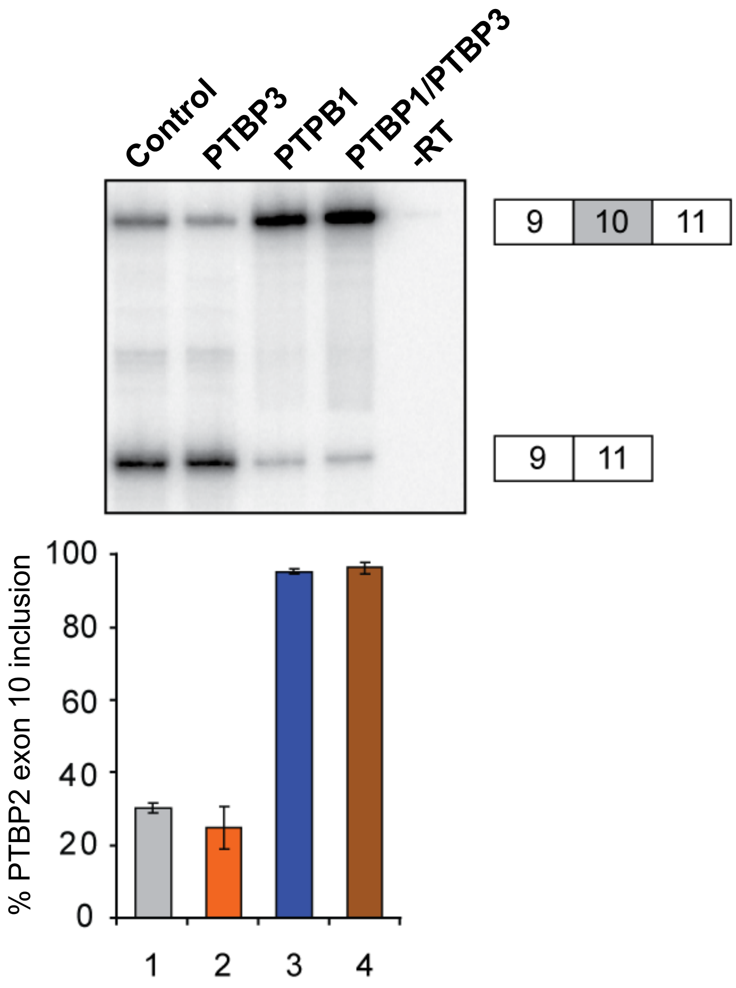

Supplementary Figure 2. Crossregulation of PTBP2 exon 10. RT-PCR of PTBP2 exon 10 splicing in K-562 cells with PTBP1 and PTBP3 knockdown. Samples were analyzed in triplicate and the averages are plotted with standard deviation error bars. The levels of exon 10 inclusion were significantly different in lanes 3 and 4 compared to lanes 1 and 2 ($p < 0.05$), but not between lanes 1 & 2 or 3 & 4.

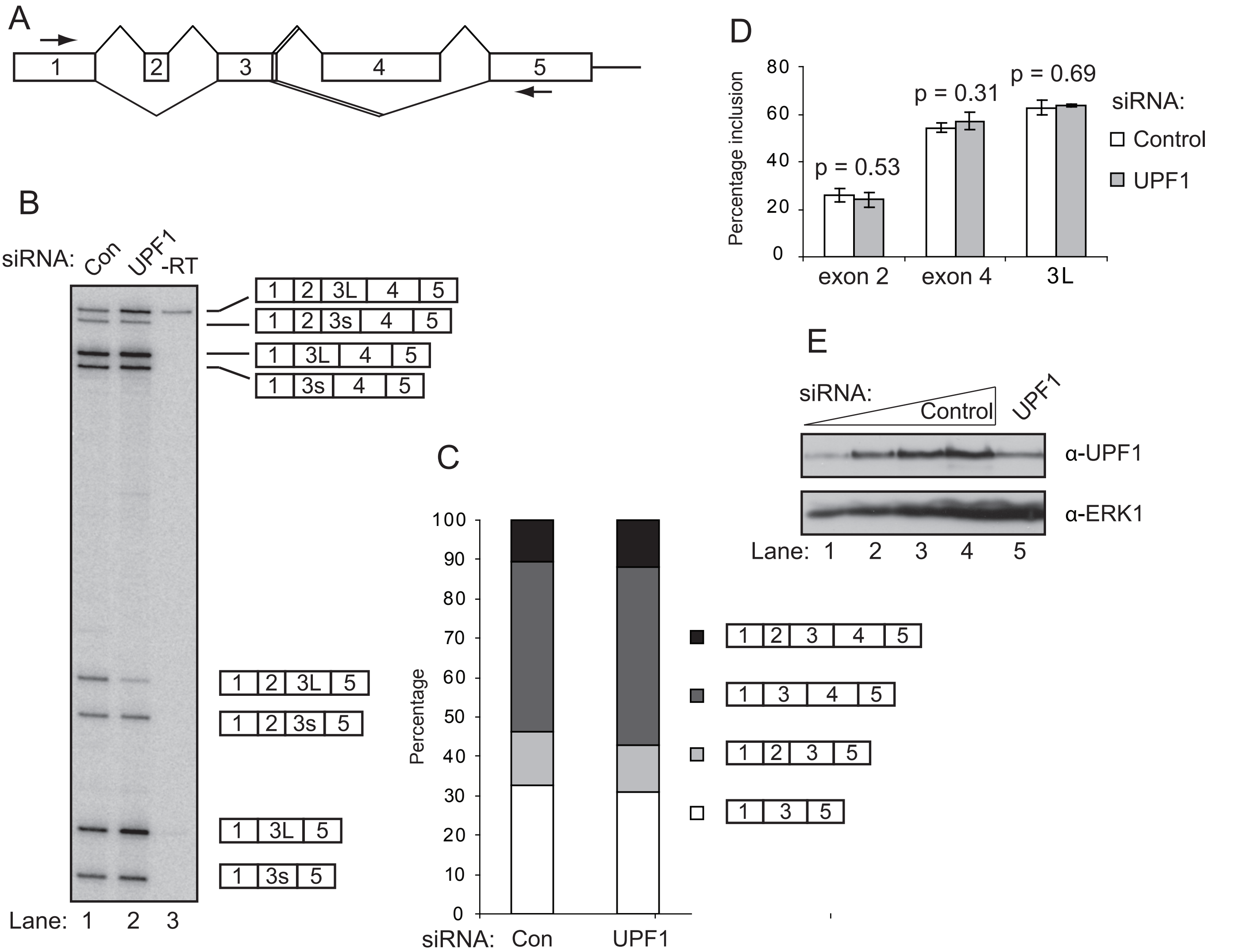

Supplementary Figure 3. *PTBP3* mRNA isoforms are not sensitive to inhibition of Nonsense Mediated Decay by *UPF1* knockdown.

A) Schematic of exon arrangement and alternative splicing events at 5’ end of *PTBP3* gene.
B) RT-PCR of *PTBP3* mRNA in K562 cells using PCR primers in exons 1 and 5 after treatment with control siRNA (lane 1) or *UPF1* siRNA (lane 2).
C) Quantitation of data from B to indicate the proportions of mRNA isoforms with exon 2 and/or exon 4 skipped.
D) Quantitation of data from B to indicate the percentage inclusion of exon 2, exon 4 and the 3L 5´ ss. None of the events are sensitive to *UPF1* knockdown.
E) Western blot of *UPF1* knockdown. Lanes, 3, 2, 1: 2-fold dilutions of the control siRNA treated sample in lane 4.

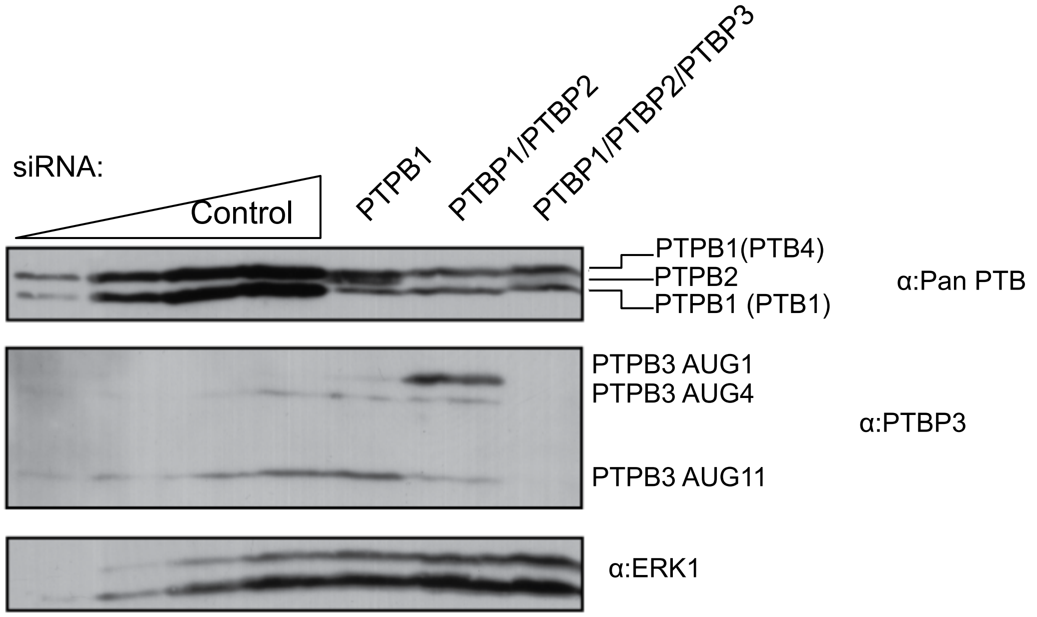

Supplementary Figure 4. Western blot analysis of HeLa cell lysates (40 µg total protein) after siRNA knockdown of combinations of PTBP1, PTBP2 and PTBP3. PTBP1 and PTBP2 were detected using a polyclonal pan-PTB antibody, PTBP3 was detected with the PTBP3 antibody and ERK1 was detected for a loading control. Control lanes contain 1:8, 1:4, 1:2, 1:1 dilutions for estimation of knockdown efficiency.

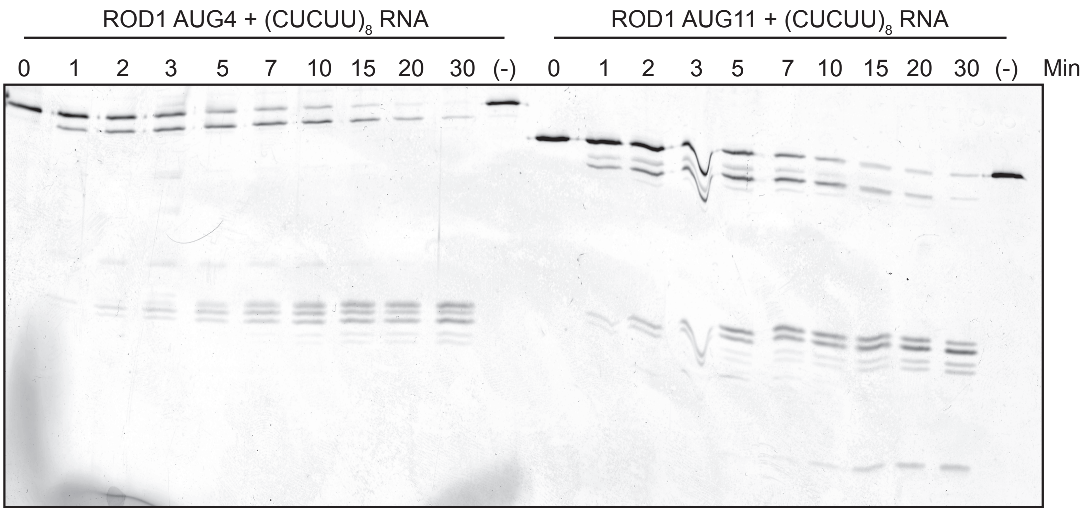

Supplementary Figure 5. Partial chymotryptic digestion of ROD1 proteins in the presence of RNA

SDS-PAGE gels comparing chymotryptic digestion patterns over a 30-minute time course for PTBP3 AUG4 and AUG11 protein in the presence of $(CUCUU)_8$ RNA at a RNA:protein molar ratio of 2:1. The (-) lane is a negative control where no chymotrypsin was added to the reaction.

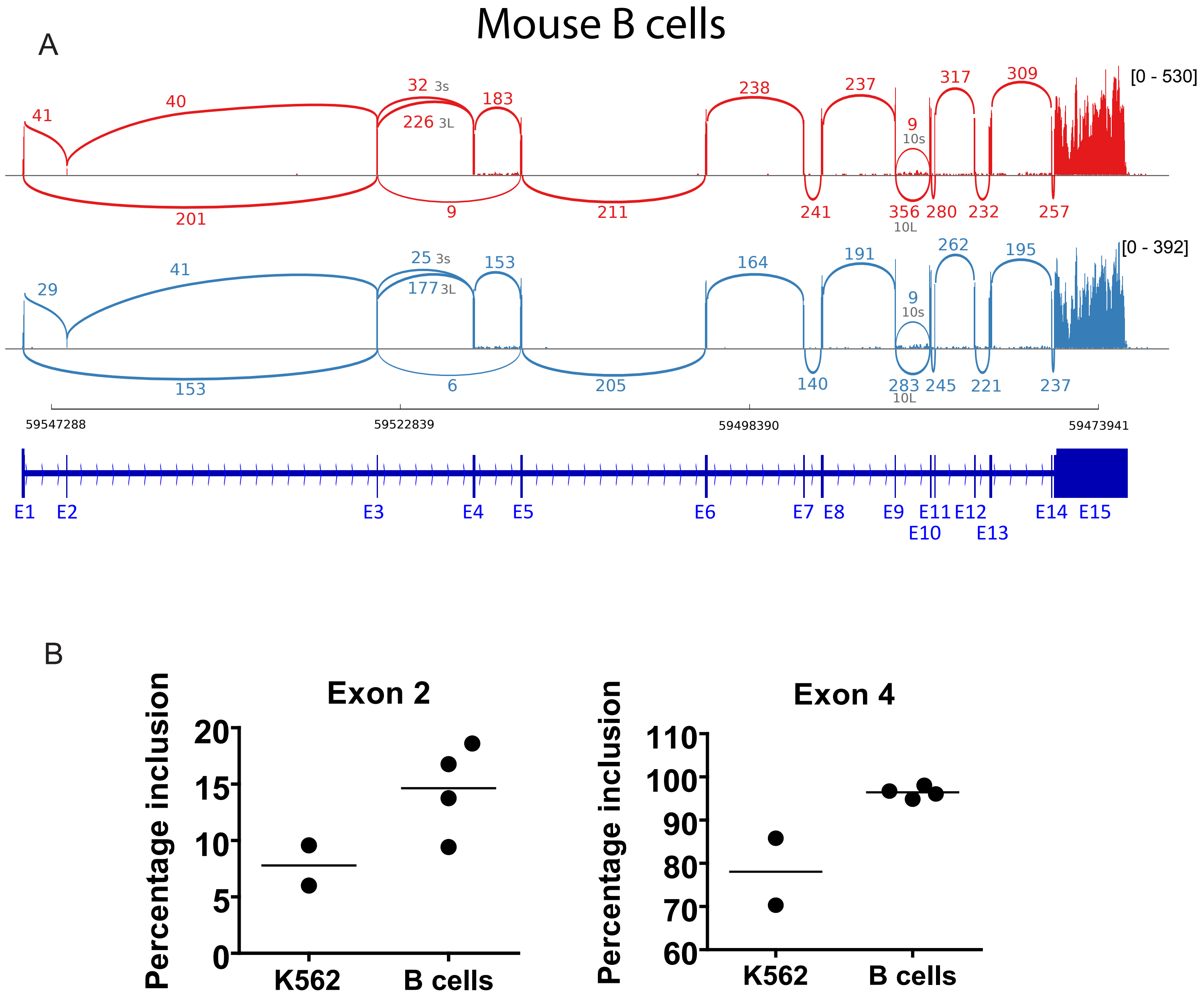

Supplementary Figure 6. Ptbp3 alternative splicing in mouse B-cells.
A) Shashimi plots for Ptbp3 of two RNAseq libraries from mouse B cells. Numbers above the arcs indicate the number of reads mapping across two exons. In addition to the alternative splicing events observed in K562 cells, two alternative 3' splice sites were observed in exon 10 separated by 15 nucleotides. Both 3' splice sites are in frame. Selection of the downstream splice site results in the deletion of a GLSVP pentapeptide in the linker between RRMs 2 and 3.
B) Quantitation from data shown in panel A (mouse B-cells) and Fig. 1A (K562 cells). Each dot shows percentage of inclusion calculated from one RNAseq library. Reads including the exon were calculated by summing the reads including the upstream and downstream exons and dividing by 2. For exon 4 no distinction was made for whether the reads were from transcripts using the upstream or downstream 5'ss of exon 3.

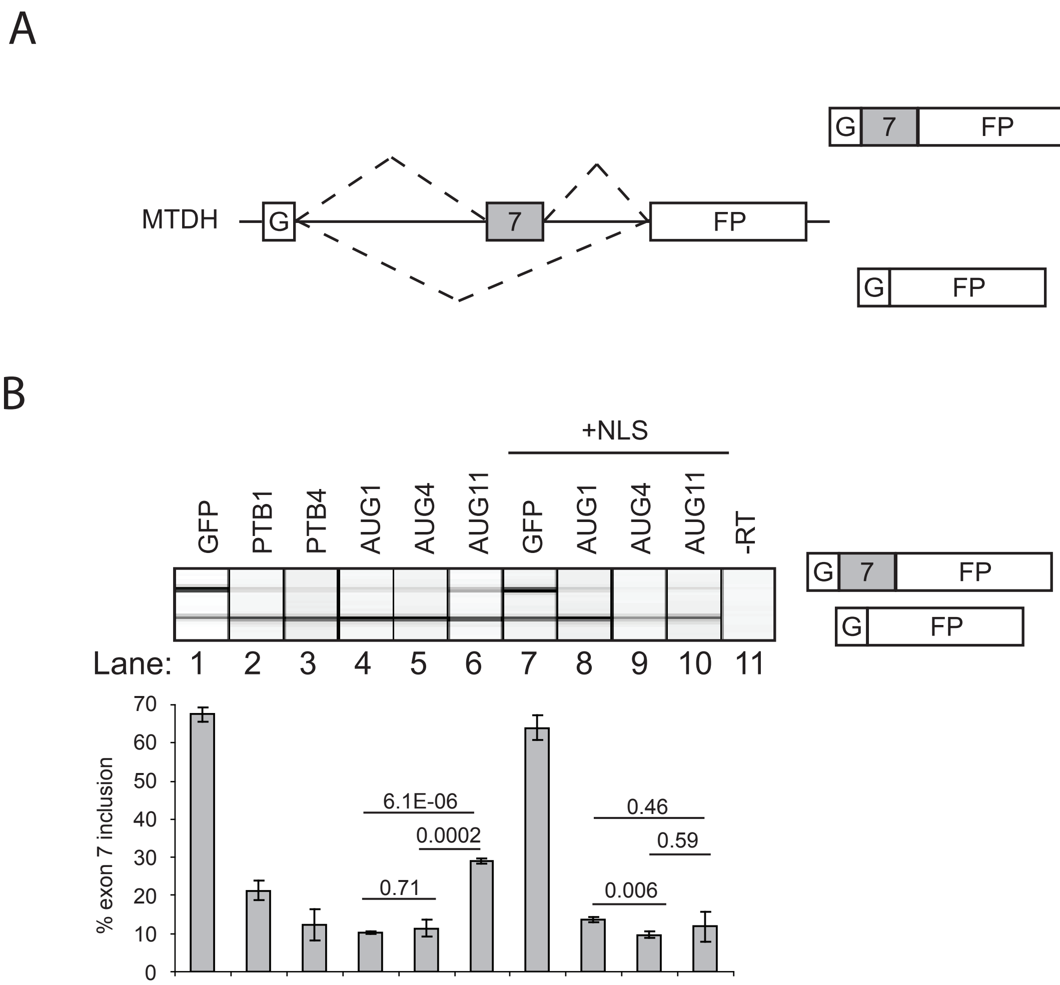

Supplementary Figure 7. PTBP3 isoforms act as repressors of MTDH exon 7

A) Schematic of MTDH exon 7 minigene.
B) Qiaxcel gel-image of RT-PCR of MTDH minigene cotransfected with GFP, as a negative control (lanes 1,7) with the PTB1 and PTB4 isoforms of PTBP1 (lanes 2, 3), and with the AUG1, AUG4 and AUG11 isoforms of PTBP3 (lanes 4-6, 8-10). Constructs in lanes 7-10 have an additional nuclear localization signal (NLS). Lane 11 is a minus reverse transcription (-RT) negative control. P-values are shown for pair-wise comparison of PTBP3 isoforms.

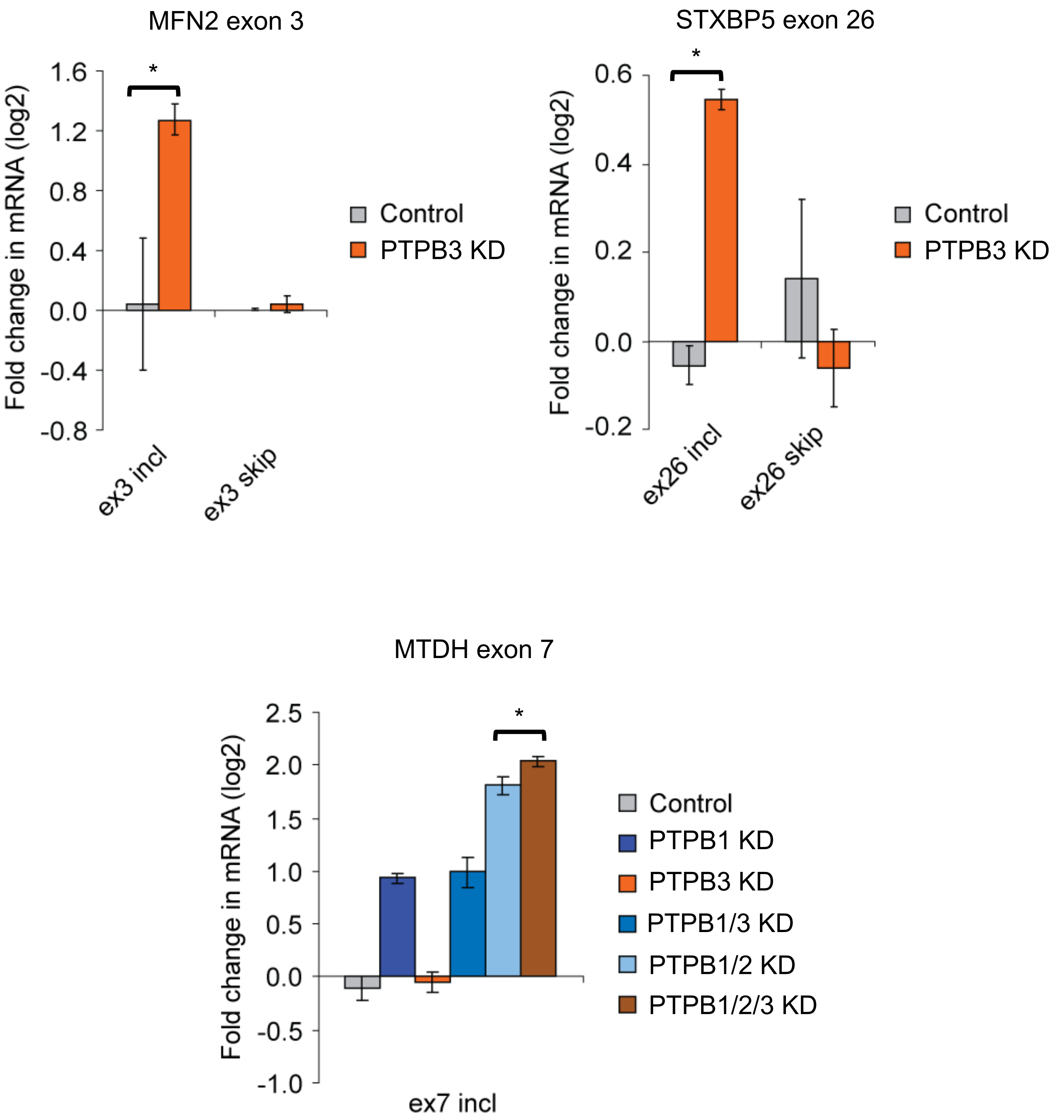

Supplementary Figure 8. Real time RT-PCR analysis of PTBP3 splicing targets in K562 cells.
A) MFN2 exon 3, B) STXBP5 exon 26, C) MTDH exon 7 inclusion
Samples were analysed in triplicate and the average mRNA levels normalized to HPRT mRNA are plotted with standard deviation error bars. To show fold change, log2 values are plotted. Asterisks indicate significant changes upon PTBP3 knockdown ($p < 0.05$).

Gene	Alternative splicing event	Cell line tested	Response to PTPB3 KD
PTBP3	Exon 2	K-562	Triple - No
MFN2	Exon 3	K-562	Single - Yes
TPM2	ME exons 6 & 7	K-562, HeLa	Triple - No
TPM2	Alt terminal exons 10 & 11	K-562, HeLa	Triple - No
ANXA7	Exon 6	K-562, HeLa	Triple - No
PKM2	ME exons 9 & 10	K-562, HeLa	Triple - No
LIMCH1	Exon 36	K-562	Single - No
MAP4K4	Exon 16	K-562	Single - No
USP5	Alt 5´ ss in exon 15	K-562	Single - No
TCF12	Exon 16	K-562	Single - No
STXBP5	Exon 26	K-562	Single - Yes
GLE1L	Exon 10	K-562	Single - No
TUBB	Alt poly A site	K-562	Single - No
MACF1	Exon 99	K-562	Single - No
MTDH	Exon 7	K-562	Triple - Yes
PDLIM5	Alt terminal exon 11	K-562	Single - No
CRYZL1	Exon 2	K-562	Single - No

Supplementary Table 1. Seventeen known PTBP1/PTBP2 regulated splicing events from Llorian et al. 2010 were tested for their response to knockdown of PTBP3 alone compared to control (“single” in column 4), or triple knockdown of PTBP1/PTBP2/PTBP3 compared to PTBP1/PTBP2 (“triple” in column 4). Only three events responded signficantly ($p < 0.05$, indicated by “Yes”), for which data are shown in Supplementary Figure 8.
